# A Motion Illusion Reveals Mechanisms of Perceptual Stabilization

**DOI:** 10.1371/journal.pone.0002741

**Published:** 2008-07-23

**Authors:** Anton L. Beer, Andreas H. Heckel, Mark W. Greenlee

**Affiliations:** 1 Institut für Psychologie, Universität Regensburg, Regensburg, Germany; 2 Experimental and Clinical Neurosciences Programme, Universität Regensburg, Regensburg, Germany; University of Southern California, United States of America

## Abstract

Visual illusions are valuable tools for the scientific examination of the mechanisms underlying perception. In the peripheral drift illusion special drift patterns appear to move although they are static. During fixation small involuntary eye movements generate retinal image slips which need to be suppressed for stable perception. Here we show that the peripheral drift illusion reveals the mechanisms of perceptual stabilization associated with these micromovements. In a series of experiments we found that illusory motion was only observed in the peripheral visual field. The strength of illusory motion varied with the degree of micromovements. However, drift patterns presented in the central (but not the peripheral) visual field modulated the strength of illusory peripheral motion. Moreover, although central drift patterns were not perceived as moving, they elicited illusory motion of neutral peripheral patterns. Central drift patterns modulated illusory peripheral motion even when micromovements remained constant. Interestingly, perceptual stabilization was only affected by static drift patterns, but not by real motion signals. Our findings suggest that perceptual instabilities caused by fixational eye movements are corrected by a mechanism that relies on visual rather than extraretinal (proprioceptive or motor) signals, and that drift patterns systematically bias this compensatory mechanism. These mechanisms may be revealed by utilizing static visual patterns that give rise to the peripheral drift illusion, but remain undetected with other patterns. Accordingly, the peripheral drift illusion is of unique value for examining processes of perceptual stabilization.

## Introduction

Whenever the eyes move, the visual scene slips across the photoreceptors of the retina. In order to obtain stable images of the world, sensory signals are suppressed during voluntary eye movements such as saccades [1]–[3]. This suppression starts briefly before saccade onset [3], and is likely initiated by efference copies of motor commands (known as ‘outflow’ theory) and maintained by proprioceptive signals (‘inflow’ theory)[1], [4].

Even when we fixate an object, the eyes show small involuntary movements such as tremors, drifts, or microsaccades [5]–[7]. These micromovements play an important role in counteracting neural adaptation [7]. However, drifts and microsaccades also cause retinal image slips that exceed the motion detection thresholds of humans [8]. Little is known about how the brain compensates for these small image slips. It is assumed that perceptual instabilities caused by involuntary eye movements are compensated by the same mechanisms that apply for voluntary saccades [7], [9]. However, periods of fixation serve to analyze the visual scene in detail. Saccadic suppression counteracts this goal as it causes widespread distortions of sensory signals [2], and starts prior to the onset of eye movements [3]. Micromovements occur involuntarily and are not controlled by motor commands. Proprioceptive signals are too imprecise [10] for adequately correcting small retinal image slips. Hence, it is possible that the mechanism that compensates for fixational eye movements relies on visual (retinal) [11] rather than extraretinal (motor commands, proprioceptive) signals [9]. Unfortunately, examining the mechanisms of perceptual stabilization has been challenging as micromovements cannot be triggered directly and - owing to its small amplitude - online detection with human eye tracking devices is unreliable.

**Figure 1:**
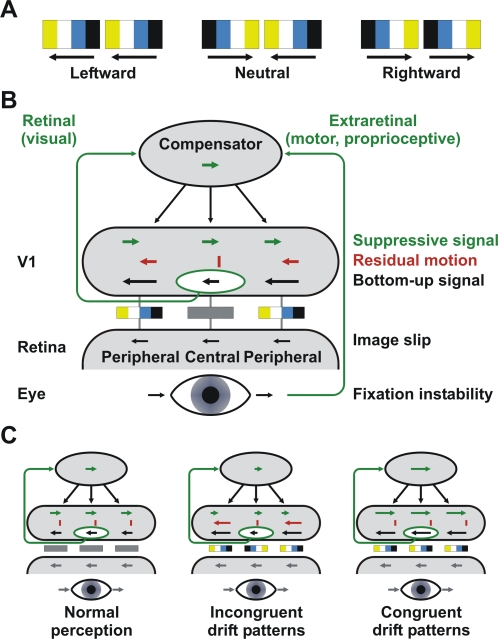
Stimuli and hypotheses. A) Drift patterns consisted of yellow, white, blue, and black vertical bars eliciting illusory motion in the black-blue to white-yellow direction [17]. For neutral patterns two adjacent patterns had opposite polarity. B) Hypothetical mechanisms for compensating micromovements. Small eye movements during fixation generate image slips on the retina. These image slips activate direction-selective cells in primary visual cortex (V1) (bottom-up). Perceptual stabilization is accomplished by suppressing eye movement evoked motion signals (suppressive signal) across the whole visual field. Eye movement velocities may be estimated based on extraretinal (motor commands, proprioceptive) signals or directly derived from retinal image slips as signaled in V1. Responses of direction-selective V1 neurons are biased consistent with the design rule of drift patterns [13], [17] and amplify or attenuate the sensation of retinal image slips. Residual motion signals resulting from the mismatch of bottom-up and suppressive signals are perceived as illusory motion. C) Hypotheses. In normal perception eye movements are veridically estimated leading to full compensation of retinal image slips. According to an extraretinal account of perceptual stabilization illusory motion should remain constant regardless of the arrangement of drift patterns. However, a retinal account predicts that illusory motion depends on the signals arising from the (central) visual field part that is used to estimate eye movements. Drift patterns in the central field bias the estimate of fixational eye movements. This bias will be opposite to the biased bottom-up signal of incongruent (with opposite polarity) peripheral patterns leading to strong illusory motion. Central drift patterns that are oriented in the same direction as peripheral patterns bias eye movement estimates in the same direction as the bottom-up peripheral motion signals leading to no or only weak illusory motion. For ease of understanding only rightward eye movements are shown. The same principles apply when the mean motion signals of isotropic eye movements are considered.

Visual illusions [12] are of unique value for neuroscience as they demonstrate failures of normal perception. Here we show that the peripheral drift illusion [12]–[18] results from a failure to compensate for micromovements. Accordingly, this illusion may be used to distinguish between retinal and extraretinal mechanisms of perceptual stabilization. In the peripheral drift illusion, static patterns in the peripheral visual field appear to move persistently in one direction. Its most effective variant - the ‘rotating snake’ illusion [16]–[18] - is composed of micropatterns containing four orderly arranged grey-scale or colored (yellow-white-blue-black) elements of different luminance (Fig. 1A). These static ‘drift’ patterns elicit illusory motion in the black-blue to white-yellow direction [17], [18]. Interestingly, neural correlates for the illusion were already found at the level of primary visual cortex in macaques [13]. Briefly flashed drift patterns elicited responses in direction-selective cells that were biased consistent with the design rule of the illusion. However, pattern onset responses may not completely explain the persistent nature of the illusion. Other studies suggested that eye movements [14], [19], particularly drift micromovements [18], contribute to the peripheral drift illusion. However, eye movements alone cannot explain why motion is perceived only with drift patterns but not in normal perception. It rather seems that the peripheral drift illusion demonstrates a failure to compensate for micromovements as illustrated in Figure 1B. If so, it would separate retinal and extraretinal mechanisms of perceptual stabilization.

Small retinal image slips during micromovements elicit directional responses in early visual cortex. In order to obtain stable perception, these motion signals need to be suppressed across the whole visual field (Fig. 1B). Static drift patterns bias the response of direction-selective cells in primary visual cortex consistent with the design rule of the illusion [13]. These biased responses cannot be fully suppressed when eye movements are veridically estimated. However, if micromovements are estimated based on visual (retinal) signals, then biased responses in primary visual cortex should also affect the magnitude of compensatory suppression. Accordingly, if the whole visual field contains drift patterns of the same polarity (congruent), then both bottom-up motion signals and estimated micromovements will be equally biased. In this case, motion responses in primary visual cortex will be fully suppressed, and the drift illusion will not appear (Fig. 1C). Conversely, if the visual field contains oppositely oriented drift patterns (incongruent), then eye movement estimates will not match the biased local motion signals in primary visual cortex. The mismatch between local motion signals and eye movement estimates will lead to inaccurate suppression and strong illusory motion. By contrast, if compensation for micromovements is solely based on extraretinal signals, the strength of the peripheral drift illusion will not vary with different pattern arrangements.

## Results

### Experiment 1

**Figure 2:**
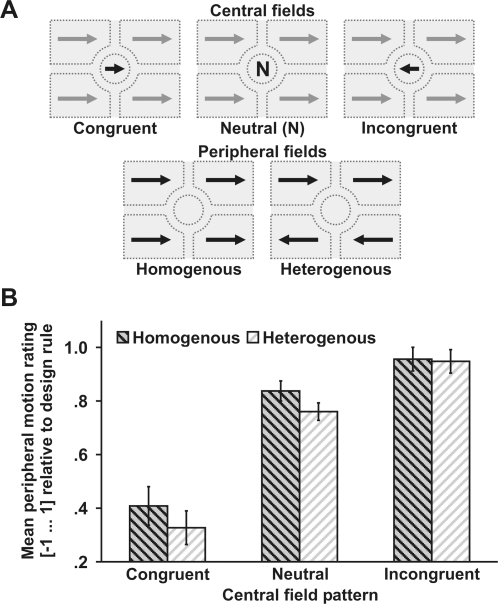
Role of central patterns (Exp. 1). A) Experimental conditions. Central field patterns were congruent (same orientation as peripheral fields), neutral, or incongruent (opposite to peripheral field patterns). Upper and lower peripheral field patterns were homogenous (same orientation) or heterogenous (opposite orientation). In separate trials observers rated motion of either the upper or lower peripheral fields. B) Perceived peripheral motion as a function of the central pattern. Illusory motion was perceived consistent with the design rule of drift patterns [17] most robustly with incongruent, intermediate with neutral, and weakest with congruent central patterns. No difference was observed between homogenous and heterogenous peripheral fields. Error bars reflect within-subjects standard errors of the mean.

In order to test this notion, we devised a new variant of the peripheral drift illusion that only contains horizontal (leftward or rightward) drift patterns (Fig. 1A). These patterns were pseudorandomly arranged within four peripheral fields (Fig. 2A). Observers were asked to direct their gaze to the middle of a circular central field (see also Supporting Video S1). In half of the trials, drift patterns in all peripheral fields had the same polarity (homogenous). The central field contained drift patterns that were either congruent (same polarity as peripheral fields), neutral (two adjacent patterns oppositely oriented), or incongruent (opposite polarity than peripheral fields). Observers rated illusory motion of peripheral fields by responding ‘leftward’, ‘no motion’, or ‘rightward’ (coded as 1 = consistent with the design rule of drift patterns; 0 = no motion; −1 = inconsistent motion), respectively. A retinal account of perceptual stabilization predicts that incongruent central patterns will lead to strong illusory motion, whereas congruent central patterns will result in no (or weak) illusory motion. Consistent with this notion observers perceived more peripheral motion with the incongruent than with the neutral central pattern, *t*(5) = 3.1, *p* = .027 (paired, two-tailed). In turn, the effect was more pronounced for the neutral compared to the congruent central pattern, *t*(5) = 4.0, *p* = .010 (Fig. 2B).

We further asked whether micromovements are estimated based on visual signals arising from the whole or just the central visual field. Therefore, in half of the trials drift patterns in the upper and lower visual field had an opposite polarity (heterogenous). A small grey rectangle presented after pattern offset indicated whether upper or lower peripheral fields had to be rated. A within-subjects ANOVA revealed a strong main effect of central field pattern, *F*(2,10) = 30.6, *p*<.001. However, the peripheral field (homogenous vs. heterogenous) had no effect suggesting that only (or predominantly) signals arising from the central visual field but not from the peripheral fields affect micromovement estimates. It may be argued that spatial attention counteracted the influence of peripheral patterns on illusory motion perception. However, the peripheral field to be judged (upper or lower) was indicated after pattern offset leaving the observers no incentives to attend one peripheral field more than others. Moreover, responses to homogenous trials were about as fast as responses to heterogenous trials (907 ms vs. 940 ms, *p*>.4). Therefore, spatial attention likely did not eliminate the influence of peripheral patterns on illusory motion. It rather seems that peripheral patterns - unlike central patterns - contribute little to perceptual stabilization.

### Experiment 2

**Figure 3:**
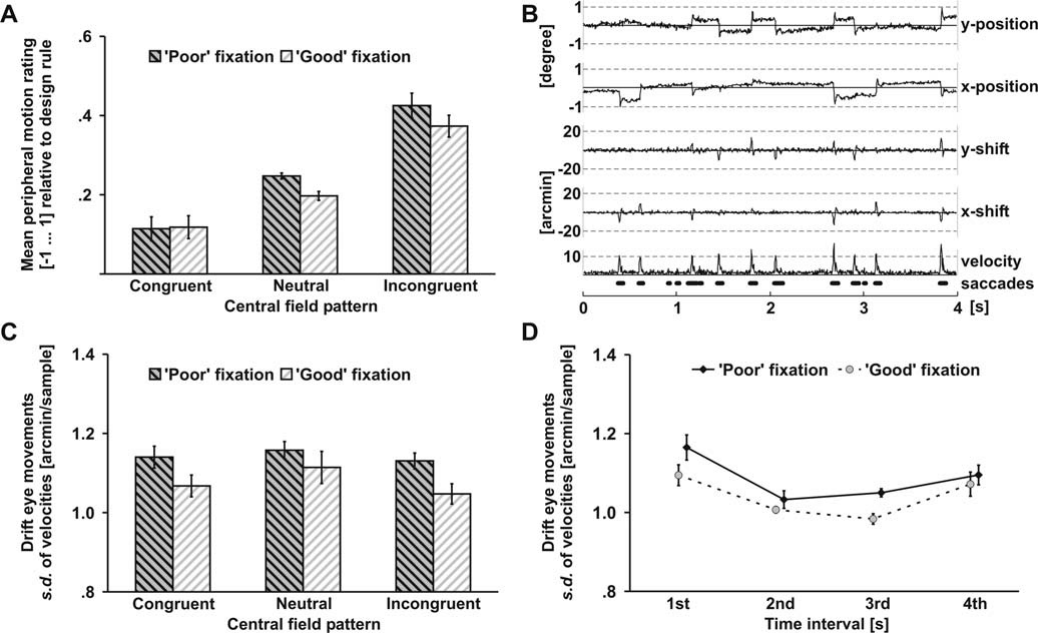
Role of eye movements (Exp. 2). A) Perceived peripheral motion varied with central patterns in both fixation conditions. The central pattern had a stronger effect in ‘poor’ (blank circle) than in ‘good’ (cross) fixation trials. B) Representative eye trajectory during pattern presentation. Four seconds (250 Hz sampling rate) of eye movements (horizontal and vertical) following pattern onset are shown. Horizontal (x) and vertical (y) eye positions were converted into instantaneous velocity vectors (shifts between two samples) and filtered by a 20 ms unweighted box-car filter. Subsequently, velocity vectors were transformed into polar vectors (direction and amplitude). Saccade periods (bold) were detected as described elsewhere [22] (see also Materials and Methods). C) ‘Poor’ fixation resulted in larger fixation instability (s.d. of instantaneous velocities of saccade-free drift periods) than ‘good’ fixation. Pattern arrangement (incongruent, neutral, and congruent) had no effect on eye movements. D) Time course of drift micromovements (fixation instability). Fixation instability was calculated for each of four consecutive time intervals (each one second) starting with pattern onset. Drift micromovements decreased between the 1st and 2nd and increased between the 3rd and 4th interval. Error bars reflect within-subjects standard errors of the mean.

The different arrangements of drift patterns may have affected the frequency and amplitude of fixational eye movements resulting in altered bottom-up signals or extraretinal signals. Therefore, we recorded eye movements while observers viewed congruent, neutral, or incongruent central fields in the context of homogenous leftward or rightward peripheral fields. Peripheral motion was rated on a five-point scale (‘strongly left’, ‘weakly left’, ‘no motion’, ‘weakly right’, or ‘strongly right’, coded from −1 to 1). Although micromovements are involuntary [7] a fixation target [20], stimulus onset [21], or attention [6] can modulate their strength. We reasoned that a ‘good’ (cross) relative to a ‘poor’ (blank circle) fixation marker reduces micromovements and causes less illusory motion. Fixation markers appeared one second prior to the drift patterns. For both fixation conditions the strength of peripheral motion (Fig. 3A) varied with the central pattern being most vivid for incongruent, intermediate for neutral, and weakest for congruent patterns, *F*(2,11) = 17.0, *p*<.001. Moreover, for incongruent and neutral centers, more motion was perceived in the ‘poor’ than in the ‘good’ fixation condition, *F*(2,22) = 3.7, *p* = .040 (interaction between central patterns and fixation conditions on z-standardized data).

Eye movement trajectories for the whole pattern duration were parsed into microsaccades and saccade-free periods (Fig. 3B) as described elsewhere [22]. Fixation instability was defined as the standard deviation of instantaneous velocities of drift micromovements during saccade-free periods. More pronounced fixation instability was observed in ‘poor’ than in ‘good’ fixation trials (Fig. 3C), *F*(1,11) = 5.8, *p* = .034. However, fixation instability did not differ for trials with congruent, neutral, or incongruent central patterns (*p*>.4). No reliable differences were observed on the mean number or mean amplitude of saccades.

Observers usually report that the peripheral drift illusion becomes less compelling after prolonged viewing [19]. Therefore, we analyzed measures of fixation instability, number of saccades, and mean saccade amplitude separately for each of four consecutive time periods (each one second) starting with pattern onset. No significant differences between central field conditions (congruent, neutral, and incongruent) were observed in any of the intervals and, hence, trials with different central patterns were combined. Consistent with the overall data, fixation instability was larger in the ‘poor’ than in the ‘good’ fixation condition as indicated by a marginally significant main effect of Fixation, *F*(1,11) = 3.7, *p* = .081 (within-subject ANOVA including the factors Fixation and Time). Additionally, a main effect of Time (1st–4th interval), *F*(3,33) = 5.5, *p* = .004, was observed (Fig. 3D). Subsequent paired t-tests (two-tailed) revealed that fixation instability was more pronounced at the first compared to the second interval, *t*(11) = 3.0, *p* = .011. Moreover, drift micromovements increased from the third to the fourth interval, *t*(11) = 2.5, *p* = .032. No reliable differences were observed on saccade measures. The decrease of fixation instability during the first second is consistent with reports showing that stimulus onset temporarily increases micromovements [21]. Alternatively, it may reflect voluntary suppression for micromovements [6] requiring some time to become effective. We did not expect an increase of drift micromovements at the end of the pattern presentation period. However, given that pattern duration was predictable (all trials lasted 4 seconds), it is possible that observers started to disengage attention from fixation prior to pattern offset in order to prepare for the subsequent response. Less attention devoted to the fixation spot may have resulted in an increase of drift micromovements [6]. Importantly, the decrease in drift micromovements corresponds well with the usual observation that the peripheral drift illusion slowly fades after prolonged fixation periods [19].

### Experiment 3

**Figure 4:**
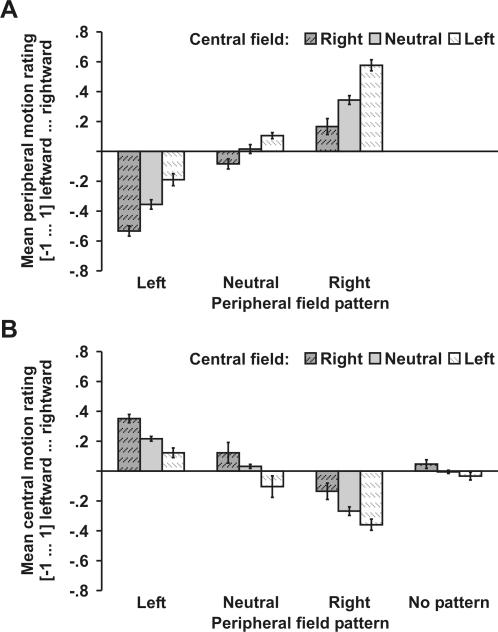
Neutral peripheral patterns (Exp. 3A) and motion of central patterns (Exp. 3B). A) Neutral peripheral patterns were perceived as moving opposite to the design rule of central patterns. B) Central patterns were perceived as moving only in the context of peripheral drift patterns. The strength of central motion varied with the strength of peripheral motion. The direction of central motion was opposite to the peripheral motion (ignoring the design rule of central drift patterns). Error bars reflect within-subjects standard errors of the mean.

Our account for the peripheral drift illusion predicts that even neutral peripheral patterns are perceived as moving when eye movement estimates are biased by central drift patterns. In Experiment 3A, observers viewed leftward, rightward, and neutral (homogenous) peripheral fields. As expected, rightward and leftward peripheral drift patterns elicited more illusory motion with incongruent than with neutral central patterns (*p*<.05), and with neutral than with congruent central patterns (*p*<.01). Interestingly, even neutral peripheral patterns were perceived as moving more leftward with a rightward than with a neutral center, *t*(7) = 2.8, *p* = .027, and more rightward with a leftward than with a neutral center (Fig. 4A), *t*(7) = 3.0, *p* = .019.

It may be argued that our findings reflect induced motion [23] caused by a moving center rather than reflecting a mechanism of perceptual stabilization. In separate blocks (Exp. 3B), observers rated the perceived motion of central fields (leftward, neutral, or rightward), which were presented together with either leftward, neutral, rightward, or no peripheral patterns. No reliable central motion was observed when the peripheral fields contained no patterns or neutral patterns (Fig. 4B). However, observers perceived central patterns as weakly moving when they were presented in the context of peripheral patterns. This motion was seen more strongly with incongruent than with neutral centers, *t*(7) = 3.4, *p* = .011, and tended to be more pronounced with neutral than with congruent centers, *t*(7) = 2.0, *p* = .086. Moreover, the direction of motion was opposite to peripheral motion regardless of the polarity of the central patterns, *t*(7) = 2.5, *p* = .043. These findings suggest that the illusory peripheral motion may induce motion in the central field. This central motion was dependent on the perceived motion in peripheral fields, but was essentially independent of the design rule of the central drift patterns. Together with the observation that no central motion was observed when peripheral patterns were absent, these findings suggest that central patterns do not evoke motion signals on their own. Accordingly, it may be inferred that central patterns are not capable of inducing peripheral motion. Instead, the illusory peripheral motion seems to occur automatically and likely reflects a failure of perceptual stabilization.

### Experiment 4

**Figure 5:**
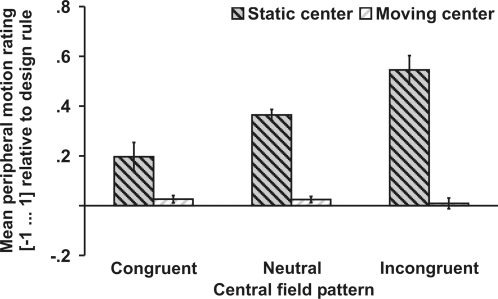
Externally versus self-induced retinal image slips (Exp. 4). Illusory peripheral motion was only perceived when the central field was static and was absent when the central field was moving. Error bars reflect within-subjects standard errors of the mean.

Finally, we tested whether the drift illusion is elicited by any retinal image slip or whether it is contingent on drift patterns and real eye movements. In half of the trials the central field was static, whereas in the other half the whole central field moved slowly in random directions. The jittering motion increases the variability of retinal slips in the central field - the region that is most important for estimating eye movements (see above). Hence, illusory peripheral motion should increase when compensation is solely based on retinal motion regardless of its origin. By contrast, the jittering center decreases the correlation between image slips and eye movements, and weaker illusory motion will be expected when retinal compensation is contingent on real eye movements. As expected, stronger illusory motion was observed for incongruent than for neutral patterns, *t*(5) = 2.7, *p* = .040, and for neutral than for congruent patterns, *t*(5) = 2.6, *p* = .049) when the center was static. However, no illusory motion was perceived when the center was moving (Fig. 5) as reflected by a main effect of central motion (static vs. moving), *F*(1,5) = 12.4, *p* = .018, and an interaction between the effect of central field patterns and the jittering central motion, *F*(1,5) = 13.5, *p* = .001.

## Discussion

Consistent with previous research [18], we found that the strength of the peripheral drift illusion varies with the degree of drift micromovements (Exp. 3). ‘Poor’ fixation resulted in a larger variability of drift micromovements and stronger illusory motion than ‘good’ fixation. Other types of eye movements, such as microsaccades, seemed to be less relevant. Fixation instability (variability of drifts) was most pronounced immediately after pattern onset and declined as fixation continued. This is consistent with the literature showing that stimulus onset temporarily increases micromovements [21], and that voluntary attention that requires some time to become effective can suppress micromovements [6]. Importantly, the decrease of fixational eye movements corresponds well with the phenomenological characteristics of the peripheral drift illusion, which is most strongly observed immediately after pattern onset and slowly fades after prolonged fixation [19].

Although micromovements are crucial for the peripheral drift illusion they may not fully explain the phenomenology of this illusion. Instead the peripheral drift illusion demonstrates a failure to compensate for retinal image slips generated by micromovements. Drift patterns presented in the central visual field modulated the strength of illusory peripheral motion (Exp. 1). Moreover, central drift patterns elicited illusory motion even on neutral peripheral patterns (Exp. 3A). None of these effects can be explained by a simple eye movement account, because central field patterns had no direct impact on the pattern of micromovements (Exp. 2). The effects of central patterns on illusory peripheral motion cannot be ascribed to ‘induced’ motion [23], in that central drift patterns elicited no motion percept when presented on its own (Exp. 3B). Our findings suggest that illusory motion results from incomplete suppression of retinal image slips that are generated by micromovements. This compensatory mechanism seems to utilize visual (retinal) signals for estimating eye movements rather than extraretinal signals [9]. Note that illusory motion was modulated by central but not peripheral drift patterns (Exp. 1) suggesting that eye movements were predominantly estimated based on visual signals from the central visual field. This central field dominance well serves the needs of visual acuity which requires images to be most stable close to the fovea.

Interestingly, only self-generated motion (eye movements) modulated illusory peripheral motion. Externally generated motion substantially reduced the percept of illusory motion (Exp. 4). With self-generated image slips retinal and extraretinal signals are strongly correlated, and the effects of retinal compensation become evident. With externally generated image slips retinal and extraretinal signals are decoupled, and retinal mechanisms of image stabilization become ineffective. This finding suggests that although extraretinal signals are too imprecise to fully compensate for micromovements [10], they may gate or modulate retinal mechanisms of image stabilization.

Failures of perceptual stabilization likely account for two other motion illusions. In the visual jitter after-effect [11], [24], observers are exposed to dynamic noise for several seconds, leading to adaptation (reduced responsiveness) of motion-selective neurons. A subsequently presented static noise pattern is perceived as randomly moving in regions nearby the adaptation stimulus, but not at the adaptation site. It was proposed that adapted neurons transmit biased (diminished) estimates of image slips to a retinal compensation mechanism. Consequently, the compensator veridically suppresses the weak motion signals in the adapted region, but fails to fully correct for motion signals outside of the adapted region. In the flicker-induced motion illusion [25], a static noise pattern encompassed by a flickering surround is perceived as moving in random directions. Both the effects of the visual-jitter after-effect and of the flicker-induced motion illusion support the general notion of a retinal compensation mechanism for eye movements. However, these illusions could not reveal whether eye movements were estimated based on signals from the central or peripheral visual field. Our findings with drift patterns (Exp. 1) suggest that the central visual field dominates. Previous accounts suggested that slow retinal image slips are compensated regardless of their origin [11], [24]. We found that only self-generated image slips contributed to illusory motion (Exp. 4), suggesting that retinal compensation most likely is gated by extraretinal signals. Previous illusions required prolonged adaptation [11], [24] or elicited unspecific effects [25]. By contrast, drift patterns bias eye movement estimates instantaneously. The direction and strength of this bias can be controlled easily.

Drift patterns were found to elicit biased responses in direction-selective cells of the primary visual cortex [13]. The classical receptive fields of these neurons are small (∼1°) [26], [27]. Our results showed that central patterns modulated the sensory experience in peripheral visual fields suggesting spatial integration in the order of about 6° or more. Potentially, long-range horizontal connections within primary visual cortex [28] or even ‘polyaxonal’ amacrine cells in the retina [29], [30] might account for such spatial integration. However, local circuits may not explain why perceptual stabilization seems to be gated by extraretinal signals (Exp. 4). Very likely, the motion signals in early visual areas are subject to top-down control from higher-level brain areas (e.g., MT or MST [31]). It may be argued that higher-level neurons assess the relative difference in motion between incongruent and congruent patterns [32] without involving early visual areas. If so, we would expect more pronounced illusory motion in heterogenous than in homogenous peripheral fields (Exp. 1). Moreover, we would expect no illusory motion for neutral peripheral patterns (Exp. 2) - given that a central drift pattern does not elicit motion. Our findings do not support a pure high-level account but are best explained by assuming feedback projections to early visual areas. Brain areas responsible for compensating micromovements likely contain direction-selective cells with relatively large receptive fields whose responses are modulated by extraretinal signals. These requirements are met by neurons in MST [33] - a subdivision of area MT+ [34]. Alternatively, area V6A [35], [36] contains neurons responsive to retinal and extraretinal signals that prefer slow speeds (<10°/s) comparable to the retinal image slips generated by drift micromovements. As cells in this area encode motion signals that are not yet corrected for eye movements - as in many other brain areas - they would be well suited for estimating self-generated retinal image slips [35].

In summary, our findings suggest that involuntary micromovements are compensated by utilizing retinal (visual) signals rather than extraretinal signals as proposed for voluntary eye movements. Drift patterns presented in the central visual field bias this retinal mechanism without affecting extraretinal signals. This type of perceptual stabilization cannot be examined with regular (neutral) patterns or real motion signals. The peripheral drift illusion seems to be of unique value for examining processes of perceptual stabilization related to small involuntary eye movements.

## Materials and Methods

### Participants

Six students (mean age 24 years, range 20–30) volunteered for experiment 1. Fifteen people were recruited for experiment 2. However, three participants had to be excluded, because they felt uncomfortable with the eye tracking arrangement (one) or eye movements could not be tracked (two). The mean age of the remaining twelve people was 29 years (range 21–42). Ten people were recruited for experiment 3. However, one person quit, because she experienced dizziness by the illusory motion. Another person reported difficulties with the task and her dataset was excluded from the analysis. The mean age of the remaining eight observers was 32 years (range 23–42). The sample for experiment 4 comprised six observers (mean age 31 years, range 23–42). All observers (except for one author in Exp. 2–4) were naive, gave written informed consent, and were compensated with 7 €/hour or credit hours. The study was approved by the ethic board of the Universität Regensburg.

### Stimuli

The drift patterns were adapted from Fraser-Wilcox Type IIa patterns [16], [17]. They consisted of black, blue, and white (<1, 15, 60 cd/m^2^) vertical adjacent bars (height .65°) on a yellow background (45 cd/m^2^) (Fig. 1A, see also Supporting Video S1). Black and white bars were .09° and blue bars were .18°, .24°, or .30° wide. The drift patterns were presented inside a circular central field (radius 4°) or peripheral fields (16°×12°) (Fig. 2A). All fields were separated by blank strips (2.3°). Within each field, patterns were evenly distributed (1.5 patterns/deg^2^) but pseudorandomly (avoiding overlaps) locally misaligned. The pattern arrangement of top, bottom, left, and right fields were mirrored in order to avoid luminance confounds. Black and white bars were exchanged in order to form leftward and rightward patterns, respectively. For neutral fields black and white bars of two adjacent patterns were alternated.

### Procedure

Observers fixated the center of a CRT (Exp. 1, 3, and 4) or LCD (Exp. 2) monitor (67 cm distant, 40×30 cm, 1152×864 pixels, 75 Hz) with their head placed on a chin rest in a dimmed room. In all experiments the patterns were presented for four seconds after which observers had to press one of three keys (‘leftward’, ‘no motion’, ‘rightward’) (Exp. 1) or one of five keys (‘strongly left’, ‘weakly left’, ‘no motion’, ‘weakly right’, ‘strongly right’) (Exp. 2–4). A small grey rectangle (1.7°×.4°) presented above, below, or to the left and right (5.15° eccentricity) indicated whether upper, lower, or central field motion, respectively, had to be judged. This indicator appeared after pattern offset in order to control for the possibility that observers preferentially attended or directed their gaze to the peripheral fields to be judged. In Exp. 1, 3, and 4 observers were asked to direct their gaze to the center of the central pattern. In Experiment 2 an additional fixation marker was provided one second prior to pattern onset consisting of a blank circle (1° radius) or a cross made of small bars (.06°×.35°) within this circle. Observers were asked to fixate inside the blank circle (‘poor’ fixation) or to precisely fixate the cross (‘good’ fixation).

In Experiment 1 (ten blocks, 48 trials each) observers were asked to judge the motion of drift patterns (leftward or rightward) presented in the upper or lower peripheral fields. Peripheral fields were either homogeneous or heterogenous (Fig. 2A). Central fields were congruent, neutral, or incongruent. Observers in Experiment 2 (4 blocks, 96 trials each) rated leftward or rightward homogenous peripheral patterns while viewing congruent, neutral, or incongruent central patterns. In half of the trials a blank circle was presented at fixation (‘poor’ fixation) in the other half a fixation cross was presented (‘good’ fixation). Eye movements were recorded for four seconds starting at pattern onset. Observers in Experiment 3 rated motion of peripheral fields in four blocks (72 trials each) and of central fields in three interleaved blocks (96 trials each). For peripheral blocks the homogeneous peripheral fields and the central fields contained leftward, neutral, or rightward patterns. In Experiment 4 (4 blocks, 96 trials each) leftward or rightward peripheral patterns had to be rated while viewing congruent, neutral, or incongruent central patterns. In half of the trials the central field was static; in the other half the central pattern moved in random directions with a speed varying (equal distribution) between 0 and 2°/s (18.75 Hz refresh rate). All stimulus conditions were pseudorandomly presented (equally likely in each block).

### Eye tracking

Eye movements (Exp. 2) were recorded (250 Hz sampling rate, nominal accuracy <.25°) with a dual infrared video eyetracker (HS Video Eyetracker, Cambridge Research Systems, Rochester, UK). Eye movement parameters were calculated for each trial and then averaged across trials. Saccades were detected (Fig. 3B) as described elsewhere [22]. Instantaneous velocity vectors were smoothed by an unweighted box-car filter (20 ms) and transformed into polar vectors. An eye-stop matrix marked samples whose instantaneous velocities dropped below 10°/s and samples showing more than 30° direction change to the previous sample. All periods between two eye-stop points whose accumulated movement amplitude exceeded 3 arcmin were classified as saccades. Drifts (fixation instability) were estimated by the standard deviation in amplitudes of the velocity vectors from saccade-free periods (excluding intervals from 12 ms before to 12 ms following microsaccades). Samples containing no measurements (e.g., due to blinks or tracking problems) were excluded. Note that this estimate includes remnant tremor movements and measurement noise (assumed to be equal across conditions) that survived data smoothing. Moreover, this procedure is conservative with respect to drift detection but tends to overclassify saccades. Trials with more than 50% invalid measurements (blinks, tracking problems) and trials containing large-scale saccades (>2°) were excluded.

## Supporting Information

Video S1 Demonstration of peripheral drift illusion. This video demonstrates the effects of central drift patterns (congruent, neutral, incongruent) on the percept of illusory motion in peripheral fields (homogenous and heterogenous) (Exp. 1).
(2.78 MB MOV)
